# Robot-assisted laparoscopic surgery using DROP-IN radioguidance: first-in-human translation

**DOI:** 10.1007/s00259-018-4095-z

**Published:** 2018-07-27

**Authors:** Philippa Meershoek, Matthias N. van Oosterom, Hervé Simon, Laurent Mengus, Tobias Maurer, Pim J. van Leeuwen, Esther M. K. Wit, Henk G. van der Poel, Fijs W. B. van Leeuwen

**Affiliations:** 1 Interventional Molecular Imaging Laboratory, Department of Radiology, Leiden University Medical Center (LUMC), Albinusdreef 2, PO Box 9600, postal zone C2-S, 2300 RC Leiden, The Netherlands; 2 Department of Urology, Netherlands Cancer Institute-Antoni van Leeuwenhoek Hospital, Plesmanlaan 121, 1066 CX Amsterdam, The Netherlands; 3 Eurorad S.A., 2 rue Ettore Bugatti, 67201 Eckbolsheim, France; 4 Department of Urology, Technical University of Munich, Klinikum rechts der Isar, Ismaninger Straße 22, 81675 Munich, Germany; 5 Martini-Clinic, University Medical Center Hamburg-Eppendorf, Martinistraße 52, 20246 Hamburg, Germany

**Keywords:** Prostate cancer, Radioguided surgery, Robotic surgery, Sentinel lymph node, Gamma probe

## Abstract

**Purpose:**

Radioguided surgery has been widely used for clinical procedures such as sentinel node resections. In the (robot-assisted) laparoscopic setting radioguidance is realized using laparoscopic gamma probes, which have limited maneuverability. To increase the rotational freedom, a tethered DROP-IN gamma probe was designed. Here we present the first in vivo feasibility study of this technology in prostate cancer patients.

**Methods:**

Ten patients scheduled for a sentinel node procedure received four injections into the prostate with (indocyanine green-)^99m^Technetium-nanocolloid and underwent preoperative imaging (lymphoscintigraphy and SPECT/CT). The DROP-IN probe was inserted via the assistant port, still permitting the insertion and usage of additional laparoscopic tools.

**Results:**

The sentinel nodes were resected using the *da Vinci*® Si robot under guidance of DROP-IN gamma tracing and fluorescence imaging. The surgeon was able to independently maneuver the DROP-IN probe using the ProGrasp® forceps of the *da Vinci*® robot and distinguish sentinel nodes from background signal (such as the injection site).

**Conclusions:**

Overall the DROP-IN design proves to be a valuable tool for robot-assisted radioguided surgery approaches.

## Introduction

Within the concept of radioguided surgery, findings in preoperative nuclear medicine imaging, e.g. scintigraphy and single-photon emission computed tomography/ computed tomography (SPECT/CT), have been successfully translated to surgery. Such radioguided resections have been explored for a range of indications, tracers and isotopes [1]. Radioguidance is most routinely applied during sentinel lymph node (SN) identification with ^99m^Tc-labeled radiocolloids (e.g. ^99m^Tc-nanocolloid) [1]. Although a wide variety of intraoperative radioguidance modalities has been studied, such as mobile gamma cameras and freehandSPECT, the gamma-ray detection probe with its acoustic and numerical feedback still remains the leading intraoperative modality [1].

Evolutionary developments in the field of surgery have increasingly pushed towards minimally invasive approaches such as (robot-assisted) laparoscopic surgery. This shift resulted in the development of laparoscopic gamma probes, which essentially are elongated gamma probes that can be inserted through a trocar. While numerous sites use such probes during laparoscopic SN procedures of, for example, cervical cancer and prostate cancer [2, 3], the probe length and its pivot point in the trocar greatly decrease the degrees of freedom (DOF) available for probe placement to four DOF vs. six in open surgery [4]. This rotational impairment limits the identification of lesions (e.g. SNs generally harbor 1% of injected dose (ID)) when located in close vicinity to high background signals (e.g. during SN procedures the injection site harbors >90% of ID) [5, 6]. Additionally, blocking a trocar with this tool denies access of other tools (e.g. suction or tissue retrieval). In case of robot-assisted laparoscopic surgery, traditional radioguidance is further complicated by the fact that the surgeon is not present at the bedside, but performs surgery via the use of a distant console. Consequently, the surgical assistant has to perform gamma tracing under verbal guidance of the surgeon.

The emergence of fluorescence imaging cameras as an integrated tool during (robot-assisted) laparoscopic procedures, with the appealing real-time visual guidance they provide, is increasingly pushing surgeons away from the field of nuclear medicine. One may argue this is a potentially dangerous phenomenon given that clinical studies with hybrid tracers such as indocyanine green (ICG)-^99m^Tc-nanocolloid, clearly indicate that fluorescence guidance cannot replace radioguidance, but rather complement it in a best-of-both-worlds scenario [7]. Nevertheless, during robot-assisted hybrid SN procedures, the above mentioned practical limitations of the laparoscopic gamma probe increasingly diminish its use during surgery. As a result, nuclear medicine findings are used for preoperative planning, fluorescence guidance for intraoperative SN identification and the laparoscopic gamma probe is generally used as back-up and ex vivo confirmatory modality [8]. To reintroduce the use of intraoperative radioguidance, and thus expand the market for (tracer) innovations in nuclear medicine, more practical laparoscopic gamma probe technologies need to become available.

Recently we reported on the development and preclinical evaluation of a tethered DROP-IN gamma probe technology [4]. In this study we report on the first-in-human experience with this technology during robot-assisted laparoscopic SN procedures of the prostate.

## Methods

### Patients

Ten patients with a Briganti nomogram-based [9] risk >5% of lymph node (LN) metastases were included. All patients were scheduled for robot-assisted radical prostatectomy (RARP), extended pelvic lymph node dissection (ePLND: all LNs around the external iliac artery and vein, the LNs within the obturator fossa, and the LNs surrounding the internal iliac artery) and SN removal. SN mapping was based on preoperative imaging (lymphoscintigraphy and SPECT/CT) acquired using either the hybrid ICG-^99m^Tc-nanocolloid [8] agent or ^99m^Tc-nanocolloid. To realize fluorescence guidance intraoperatively in the latter group free ICG (5 mg/mL ICG) was injected transperineal intrapostatic, approximately 15 min before the start of the surgical procedure. Informed consent was obtained from all individual participants included in the study. The study was approved by the local ethics committee (NL57838.031.16). Patient characteristics are shown in Table 1.

**Table 1:** Summary findings

Characteristic	Value
Patients included	10
Age (years; mean ± SD (range))	68.9 ± 5.7 (59–77)
Patients receiving tracer(s) (%)	
- ICG-^99m^Tc-nanocolloid	- 5 (50%)
- ^99m^Tc-nanocolloid + ICG	- 5 (50%)
Total SN related hotspots on SPECT/CT	30
- Left	- 16
- Right	- 14
Total SNs specimens excised	35
- Left	- 18
- Right	- 17
Total SNs measured in vivo with DROP-IN probe	20
- **Externa right** (%)	- **3 (15%)**
- **Interna right** (%)	- **0 (0%)**
- **Obturator right** (%)	- **4 (20%)**
- **Cloquet right** (%)	- **1 (5%)**
- **Presacral left** (%)	- **1 (5%)**
- Other locations (%)	- 11 (55%)
In vivo radioactivity	
- DROP-IN median counts (range))	675 (200–2600)
Total SNs measured in vivo for fluorescence	19^a^
- Fluorescent SNs (%)	- 19 (100%)
Ex vivo radioactivity	
- DROP-IN probe (median counts (range))	682 (89–7000)
- Neoprobe (median counts (range))	378 (33–14,000)
Total SNs measured ex vivo for fluorescence	18^a^
- Fluorescent SNs (%)	- 18 (100%)
Pathological examination (%)	
SNs found in excised SN specimens	60 (100%)
- SNs tumor positive	- 1 (1.7%)
LNs found in excised nonSN specimens	149 (100%)
- LNs tumor positive	- 0 (0%)
Resection status per patient (*n* = 10)	
R0	6 (60%)
R1	4 (40%)
Postoperative complications (%)	
Yes	6 (60%)
- Urinary retention	- 2 (20%)
- Urinary leakage	- 2 (20%)
- Urinary infection	- 1 (10%)
- Parastomal hernia	- 1 (10%)
No	4 (40%)

^a^All SNs that were measured for fluorescence were fluorescent in- and ex-vivo. One patient did not receive free ICG, and in one patient the SN was not measured ex vivo

### Surgical (imaging) tools

For the robot-assisted surgical procedure the *da Vinci*® Si (Intuitive Surgical Inc., Sunnyvale, CA, USA) with integrated *Firefly*™ fluorescence laparoscope (Intuitive Surgical Inc.) was used. For gamma tracing, a DROP-IN probe prototype (Eurorad S.A., Eckbolsheim, France) was used [4]. A sterile DROP-IN was inserted into the abdominal cavity either through or next to a trocar within the Alexis® port (Alexis laparoscopic system, Applied Medical Corp., Rancho Santa Margarita, CA, USA).

### Surgical procedure

**Fig. 1:**
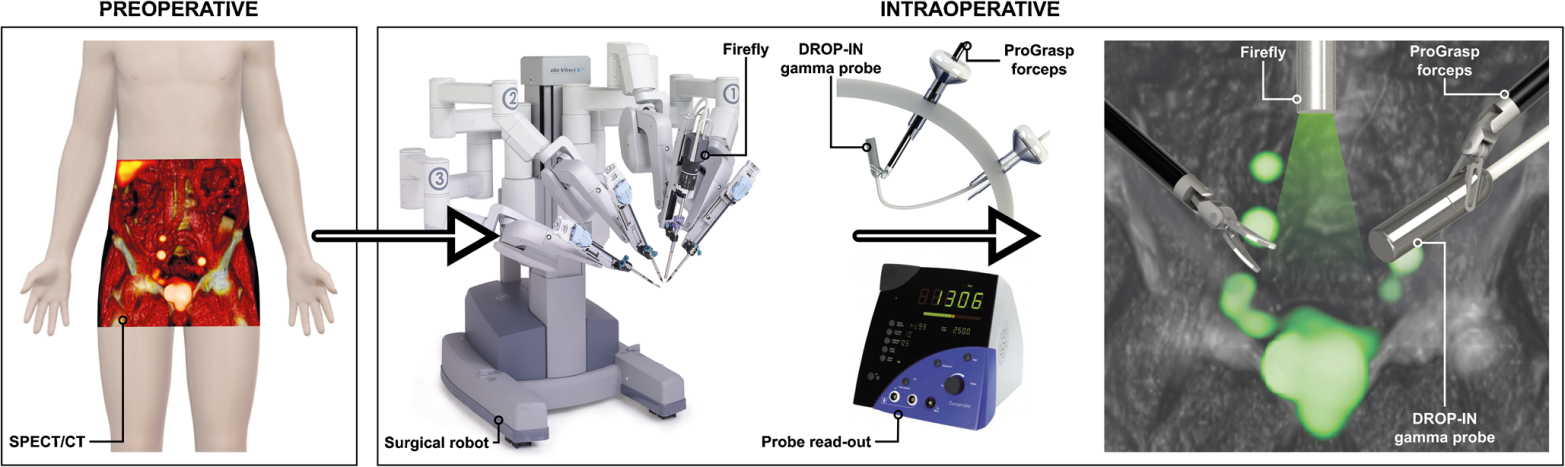
Schematic DROP-IN concept. Going from preoperative imaging (SPECT/CT) to intraoperative guidance using a combination of the DROP-IN gamma probe and the Firefly fluorescence laparoscope integrated in the da Vinci surgical robotic system

SPECT/CT images provided a roadmap wherein the tracer accumulating SNs were displayed in their anatomical context (Fig. 1) [8]. (Suspected) SN locations were then intraoperatively scanned with the DROP-IN, while the laparoscope was frequently switched between white light and fluorescence imaging. When SNs were located in a region with a high risk of surgical complications, the decision was made to leave these SNs in situ. The number and location of pre-, intra-, and post-operatively identified SNs were recorded, including fluorescent and radioactive status (Table 1). Prostatectomy followed the nodal dissection (both SN and ePLND).

Ex vivo, a conventional gamma probe (Neoprobe, Devicor Medical Products, Cincinnati, OH, USA) and an open surgery fluorescence camera (FIS-00 or PDE; Hamamatsu Photonics K.K., Hamamatsu, Japan) was used to validate tracer uptake.

## Results

### Intraoperative sentinel node identification

SPECT/CT revealed 30 SN-related hotspots and during surgery 35 SNs were excised using a combination of intraoperative DROP-IN radioguidance and fluorescence guidance (Table 1).

**Fig. 2:**
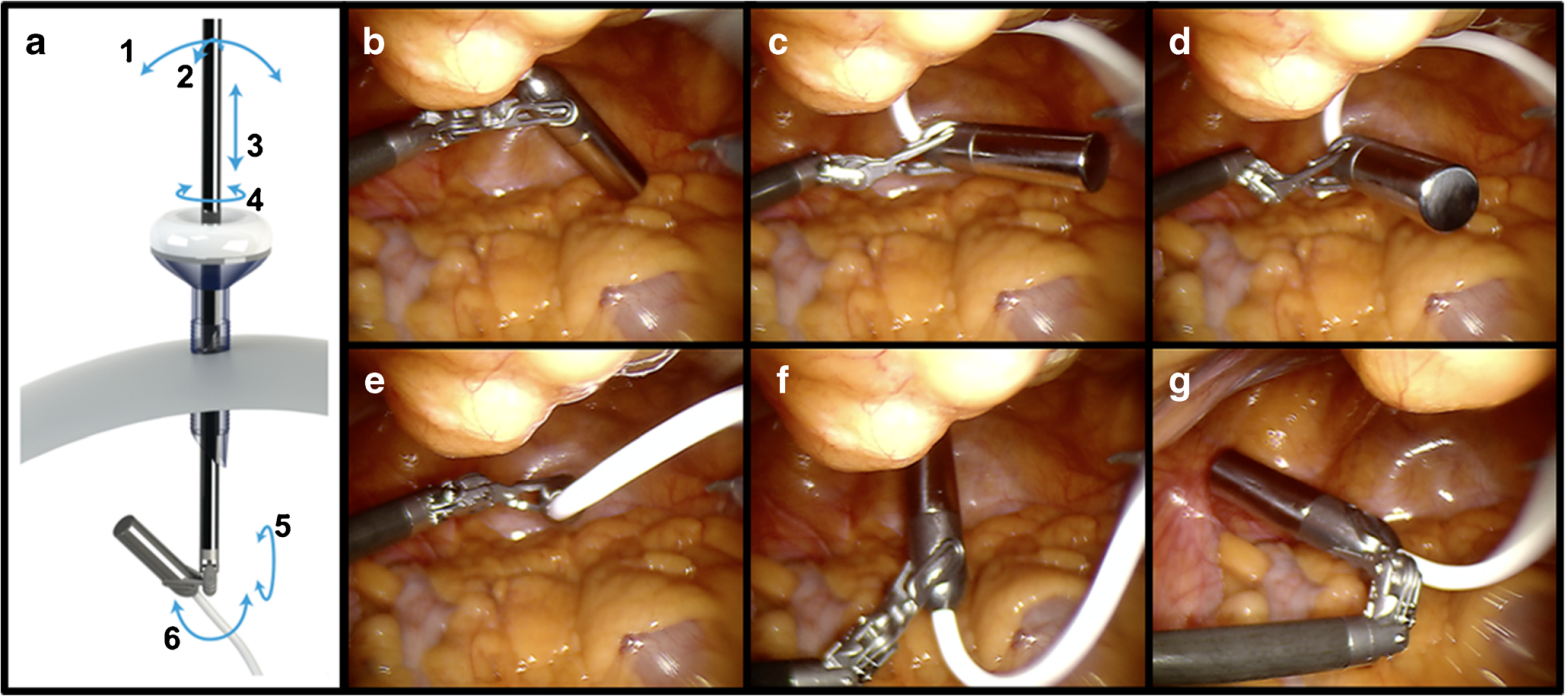
Rotational freedom of the DROP-IN probe in vivo. (**a**) The *da Vinci*® ProGrasp® forceps was inserted from the lower left abdominally placed trocar. *Arrows* indicate the degrees of freedom. (**b**–**g**) The small gamma probe head connected to a flexible wire resulted in freely maneuvering the probe by the surgeon using the forceps

**Fig. 3:**
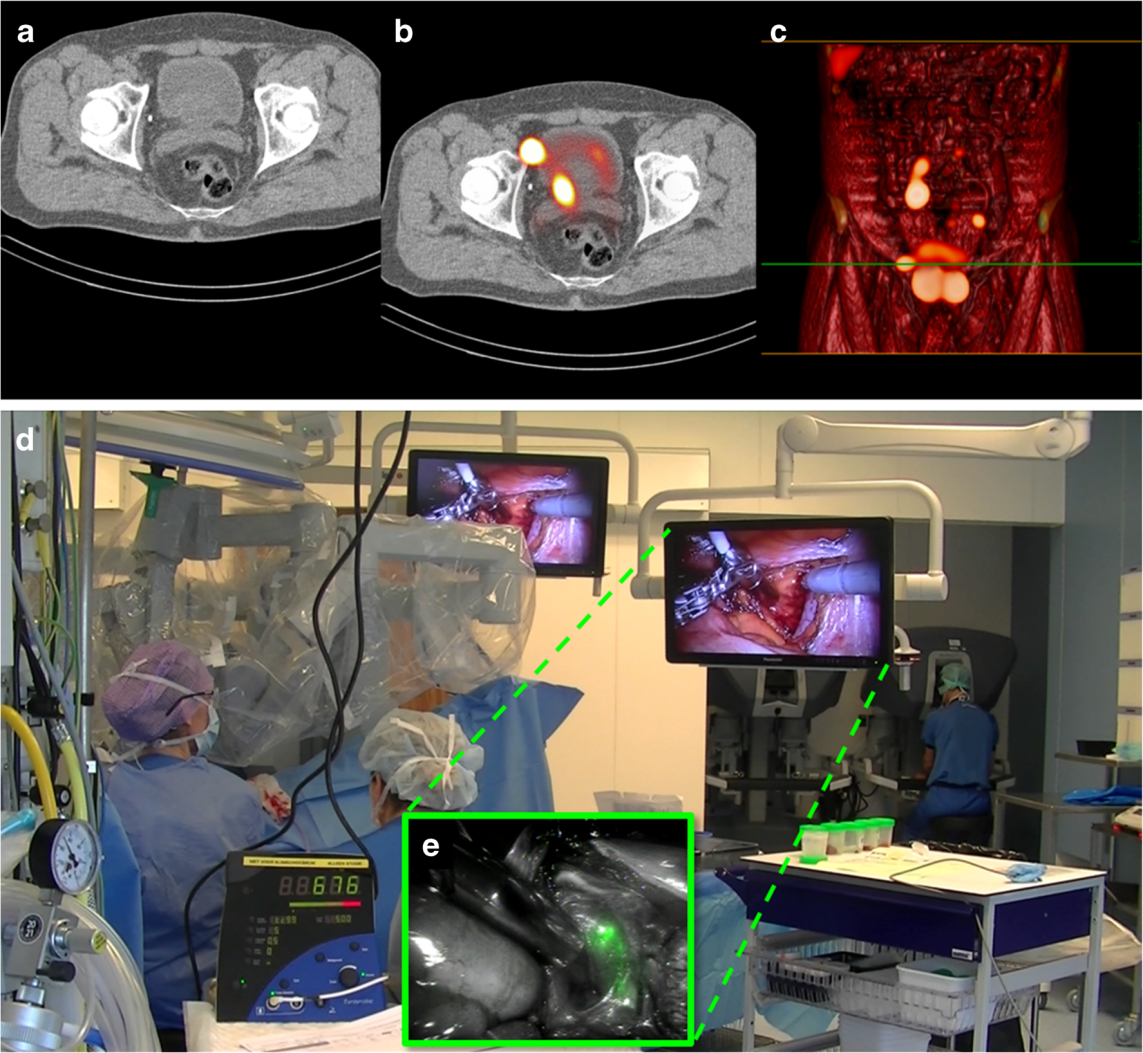
The surgical context of robot-assisted sentinel lymph node procedures—DROP-IN vs. fluorescence. Preoperative (**a**) CT, (**b**) SPECT/CT and (**c**) a volume rendered SPECT/CT give the surgeon information about the anatomical location of the sentinel nodes (SNs). (**d**) The surgeon operates the robotic console distant from the patient bed (in the background), but autonomously positions the DROP-IN probe towards a SN. The DROP-IN console provides a radioactive count number and acoustic signal as read-out. (**e**) As insert the *Firefly*™ fluorescent image of the SN identified using the DROP-IN

Through the combination with the Alexis® port, the DROP-IN probe could remain in the patient during the complete nodal resection, while the surgical bedside assistant could still use a grasper through the assistant trocar. With the articulated ProGrasp® forceps probe pick-up and placement could be realized without interference of the surgical assistant. Probe positioning was considered intuitive and benefitted from a wide scanning range (0°–140°) (Figs. 2 and 3). A total of 45% of the removed SNs that were measured with the DROP-IN in vivo were located in regions classified as difficult for traditional laparoscopic gamma tracing: external, internal, obturator and cloquet region right, and presacral region left (Table 1). As the study was performed using both fluorescent and radioactive signals, the use of the DROP-IN was benchmarked against using the *Firefly*™ technology (Fig. 3).

No side effects were observed while using the DROP-IN intraoperatively. Postoperative complications did occur (Table 1), however, they were not related to the use of the SN-procedure or DROP-IN probe, indicating it is safe to use.

### Pathological examination

From the 35 excised SN specimens one tumor positive SN was recovered (Table 1). The excised ePLND templates (excision of 149 additional LNs) did not yield positive LNs.

## Discussion

Our feasibility study in prostate cancer patients underlines the ability of the DROP-IN technology to freely scan every direction in the abdominal cavity and supports the symbiotic use of gamma tracing and fluorescence imaging. The accuracy and convenience created by the DROP-IN technology increases the surgical implementation of nuclear medicine findings. In particular, it facilitates radioguidance concepts during (robot-assisted) laparoscopic surgery.

The difference between the number of SN-related hotspots and amount of excised SNs is most likely a consequence of cluster nodes. Previously we found that during nodal resections one hotspot on SPECT/CT often contains multiple LNs that only become distinguishable using intraoperative fluorescence guidance or following pathological examination of the excised tissue [8].

To date, radioguidance has proven itself to be the most sensitive surgical guidance modality, allowing gamma tracing even when fluorescence is not detectable [6]. Hence, in addition to advancing laparoscopic SN procedures, the DROP-IN probe technology also holds future promise for receptor targeted radioguidance procedures. An obvious example of such an emerging radioguidance procedure that could benefit from the DROP-IN to make it compatible with, for example, the robotic *da Vinci*® platform would be the tracing of prostate specific membrane antigen (PSMA) expressing nodal metastases using ^99m^Tc-PSMA-I&S [10]. As robot-assisted surgery is rapidly advancing beyond urology, likely the DROP-IN technology will support the translation/implementation of radioguidance in other indications.

## Conclusion

Our clinical first-in-human feasibility study indicates the DROP-IN: (1) facilitated radioguided resections, (2) increased the autonomy of the operating surgeon, (3) enlarged the probe maneuverability and (4) left the assistant trocar free for the introduction of other surgical tools. Further studies aimed at evaluating the technology in the clinical setting, including ^99m^Tc-PSMA-I&S guided procedures, are ongoing.
